# Daily Oral Supplementation with 60 mg of Elemental Iron for 12 Weeks Alters Blood Mitochondrial DNA Content, but Not Leukocyte Telomere Length in Cambodian Women

**DOI:** 10.3390/nu13061877

**Published:** 2021-05-31

**Authors:** Shannon L. Steele, Anthony Y. Y. Hsieh, Izabella Gadawski, Hou Kroeun, Susan I. Barr, Angela M. Devlin, Hélène C. F. Côté, Crystal D. Karakochuk

**Affiliations:** 1 Food, Nutrition and Health, University of British Columbia, 2205 East Mall, Vancouver, BC V6T 1Z4, Canada; shannon.steele@ubc.ca (S.L.S.); susan.barr@ubc.ca (S.I.B.); 2 BC Children’s Hospital Research Institute, 938 West 28th Ave, Vancouver, BC V5Z 4H4, Canada; adevlin@bcchr.ca; 3 Department of Pathology and Laboratory Medicine, University of British Columbia, 2211 Wesbrook Mall, Vancouver, BC V6T 1Z7, Canada; anthony_y_hsieh@hotmail.com (A.Y.Y.H.); izabelle@mail.ubc.ca (I.G.); hcote@pathology.ubc.ca (H.C.F.C.); 4 The Centre for Blood Research, University of British Columbia, 2350 Health Sciences Mall, Vancouver, BC V6T 1Z3, Canada; 5 Helen Keller International, Street 476, Phnom Penh 12301, Cambodia; hkroeun@hki.org; 6 Department of Paediatrics, University of British Columbia, 4480 Oak Street, Vancouver, BC V6H 3V4, Canada

**Keywords:** iron, supplementation, leukocyte telomere length, mitochondrial DNA content, oxidative stress, cellular damage, Cambodia, women

## Abstract

There is limited evidence regarding the potential risk of untargeted iron supplementation, especially among individuals who are iron-replete or have genetic hemoglobinopathies. Excess iron exposure can increase the production of reactive oxygen species, which can lead to cellular damage. We evaluated the effect of daily oral supplementation on relative leukocyte telomere length (rLTL) and blood mitochondrial DNA (mtDNA) content in non-pregnant Cambodian women (18–45 years) who received 60 mg of elemental iron as ferrous sulfate (*n* = 190) or a placebo (*n* = 186) for 12 weeks. Buffy coat rLTL and mtDNA content were quantified by monochrome multiplex quantitative polymerase chain reaction. Generalized linear mixed-effects models were used to predict the absolute and percent change in rLTL and mtDNA content after 12 weeks. Iron supplementation was not associated with an absolute or percent change in rLTL after 12 weeks compared with placebo (ß-coefficient: −0.04 [95% CI: −0.16, 0.08]; *p =* 0.50 and ß-coefficient: −0.96 [95% CI: −2.69, 0.77]; *p =* 0.28, respectively). However, iron supplementation was associated with a smaller absolute and percent increase in mtDNA content after 12 weeks compared with placebo (ß-coefficient: −11 [95% CI: −20, −2]; *p* = 0.02 and ß-coefficient: −11 [95% CI: −20, −1]; *p*= 0.02, respectively). Thus, daily oral iron supplementation for 12 weeks was associated with altered mitochondrial homeostasis in our study sample. More research is needed to understand the risk of iron exposure and the biological consequences of altered mitochondrial homeostasis in order to inform the safety of the current global supplementation policy.

## 1. Introduction

Anemia is defined as a hemoglobin concentration <120 g/L in non-pregnant women of reproductive age [1]. Adverse consequences of anemia include poor maternal health and birth outcomes [2] and reduced work productivity [3]. In 2011, it was reported that ~29% of non-pregnant women worldwide had anemia [4]. In response, the World Health Organization (WHO) set a global nutrition target to achieve a 50% reduction in anemia among women of reproductive age by 2025 [4]. As iron deficiency is thought to be the predominant cause of anemia globally [1,5], the WHO recommends untargeted daily iron supplementation with 60 mg of elemental iron for three consecutive months per year for all women and adolescent girls in regions where anemia prevalence is ≥40% [6].

In Cambodia, ~43% of non-pregnant women of reproductive age have anemia [1]. Although the prevalence of anemia among Cambodian women is high, recent reports suggest that the prevalence of iron deficiency, based on ferritin concentrations ≤15 µg/L, is much lower (≤10%) [7,8,9,10]. If so, untargeted daily oral iron supplementation would likely be ineffective and/or a waste of resources. Anemia in this population could be the result of other factors, such as micronutrient deficiencies [11], inflammation [12,13], and genetic hemoglobinopathies [14]. In Cambodia, hemoglobinopathies (e.g., thalassemia or hemoglobin E [HbE] disease) are common and affect >50% of the population [7,9,15].

There is strong evidence that iron supplementation is effective in treating women who are iron-deplete (i.e., ferritin <15 µg/L) [16]. However, evidence regarding the safety of iron supplementation in women who are iron-replete is limited. Excess iron has the potential to be harmful, as free iron can promote the formation of reactive oxygen species (ROS) via the Fenton reaction [17,18,19]. In turn, ROS can cause cellular damage, including to membrane lipids and DNA [20,21]. Of concern, women with certain genetic hemoglobin disorders, such as those with HbE homozygosity or HbE/β-thalassemia heterozygosity, have altered iron metabolism and are thus at greater risk for excess iron accumulation [9,22]. Thus, it is particularly important to determine the possible side effects of iron supplementation in settings like Cambodia, where the risk of excess iron accumulation and the prevalence of genetic hemoglobinopathies are high.

Glutathione is an abundant antioxidant that plays a critical role in protecting cells against oxidative damage [23]. The ratio of reduced glutathione (GSH) to oxidized glutathione (GSSG) is commonly used to indicate oxidative stress at the cellular level, with lower ratios indicating more oxidative stress [24]. Evidence suggests that iron supplementation can lead to a significant increase in GSSG, demonstrating its ability to cause oxidative imbalance [25]. Furthermore, previous research has suggested an association between oxidative stress and decreased leukocyte telomere length (LTL) [26] as well as altered (i.e., increased or decreased) mitochondrial DNA (mtDNA) content [27,28]. Additionally, associations between shorter telomere length and several age-related diseases, such as cardiovascular disease and cancer, have been observed [29,30]. Telomere attrition typically occurs gradually in adults, although the inter-individual variability is high [31]. Nevertheless, studies have detected changes in LTL in response to various interventions (e.g., calorie-restricted diet resulting in weight loss, exercise, and meditation) and pregnancy in less than 12 weeks [32,33,34,35]. There remains an extensive gap in the literature regarding short-term changes in telomere length. In contrast, mitochondria are well known for their highly dynamic nature, often resulting in rapid mtDNA content changes to maintain mitochondrial homeostasis alterations in mtDNA [36]. Mitochondrial dysfunction is closely associated with ROS generation and oxidative stress in cells, and an association between GSH, GSSG, and mtDNA has been previously reported in myoblasts [37]. A recent review suggests that lower mtDNA content is associated with a number of adverse health conditions, although findings were variable [38]. This variability may be explained by the dynamic nature of mitochondrial homeostasis, in which oxidative stress-induced damage to mtDNA can lead to mitophagy or mitochondrial biogenesis [36], leading to decreased or increased mtDNA content, respectively. Over the short term, both mtDNA content alterations highlight the potential inability to maintain mitochondrial homeostasis, which could ultimately impact mtDNA content in the long term. As these dynamic changes can occur quite rapidly, mtDNA content is a suitable biomarker for observing short-term effects on mitochondrial homeostasis [39].

First, we conducted a pilot study to measure the GSH/GSSG ratio, an indicator of oxidative stress, in *n* = 69 randomly selected women who received iron versus placebo. The results from this pilot study provided the impetus to further investigate markers of cellular damage in the larger study sample (*n* = 376). Thus, we evaluated the effect of daily oral supplementation with 60 mg of elemental iron for 12 weeks, compared with placebo, on two biomarkers of cellular damage: relative LTL (rLTL) and absolute mtDNA content in *n* = 376 Cambodian women.

## 2. Materials and Methods

### 2.1. Study Design

**Figure 1:**
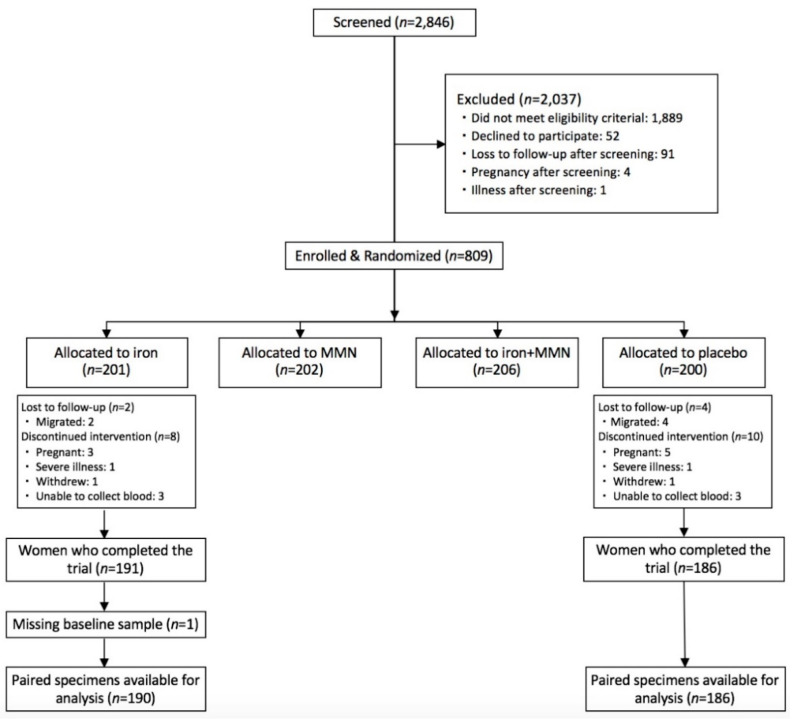
Flow chart of enrolment from the trial for women whose specimens were included in the current study. MMN, multiple micronutrients.

We used venous blood samples that were previously collected in a 2 × 2 factorial double-blind randomized controlled trial in Cambodia [40]. Ethics approval was obtained from the University of British Columbia Clinical Research Ethics Board in Canada (H15-00933) and the National Ethics Committee for Health Research in Cambodia (110-NECHR). The trial was registered at clinicaltrials.gov (NCT-02481375). The complete design, protocol, and methodology have been previously published [40]. Non-pregnant women (*n* = 809) aged 18–45 years and pre-screened as anemic were randomized to receive daily oral iron (60 mg elemental iron as ferrous sulfate; iron group), 14 other micronutrients not including iron (multiple micronutrient (MMN) group), iron + MMN, or maltodextrin capsules (placebo group). Capsule counts were conducted at 4, 8, and 12 weeks to monitor adherence. Women were considered adherent if they consumed ≥80% of the capsules, based on the average of the three capsule counts. For the pilot study, the GSH/GSSG ratio was determined in a random subgroup of *n* = 69 women. In the secondary analysis reported herein, rLTL and mtDNA contents were measured in baseline and 12-week buffy coat specimens from all women in the iron (*n* = 190) and placebo (*n* = 186) groups who completed the trial and had paired baseline and 12-week specimens available for analysis. Figure 1 presents the flow of trial enrolment. Ethics approval for this secondary analysis was obtained from the University of British Columbia Research Ethics Board (H17-02650).

### 2.2. Blood Collection and Processing

Phlebotomists collected a morning fasting venous blood sample at baseline and 12 weeks. Blood was collected in a trace element-free tube and two evacuated tubes containing EDTA (Becton Dickinson, Franklin Lakes, NJ, USA). A covered icebox was used to transport the blood to the National Institute of Public Health Laboratory in Phnom Penh, Cambodia for processing within 2–4 h of collection. Plasma, serum, and buffy coat were stored at −80 °C in 2 mL vials prior to shipment on dry ice to Canada or other laboratories for analysis. DNA was extracted from buffy coat using a QiaAmp DNA Blood Mini kit (Qiagen Ltd., Hilden, Germany) and then frozen at −80 °C for approximately 3 years. Full details of these methods have been published elsewhere [40]. DNA extracted with this method and frozen at −20 °C has been shown to be stable for long-term storage [41]; thus, there were no concerns regarding the stability of the biomarkers of cellular damage.

### 2.3. GSH/GSGG Determination

Levels of GSH and GSSG were determined in plasma using the DetectX^®^ Glutathione (GSH) Fluorescent Detection Kit (Arbor Assays, Ann Arbor, MI, USA), according to the manufacturer’s instructions. This assay measures the concentration of GSH and total glutathione, and the concentration of GSSG is calculated based on these values.

### 2.4. rLTL and mtDNA Content Determination

Monochrome multiplex quantitative polymerase chain reaction (MMqPCR) assays were used to measure both rLTL and mtDNA content in DNA extracted from buffy coat, as described by Hsieh et al. [42,43]. Briefly, DNA specimens were randomized and assayed in duplicate using the LightCycler480 platform, and data were acquired with software version 1.5.1.62 SP2. To prepare the LightCycler^®^ 480 96-well plate, 8 μL of assay mix and 2 μL of diluted DNA specimen were pipetted into each well, with baseline and 12-week specimens in adjacent wells on the same plate. A standard curve was included in the center of the plate. The plate also contained two positive internal controls (ICs) and a negative control, included in duplicate on each plate. For the rLTL assay, the positive ICs consisted of a long telomere IC (DNA extracted from pooled whole blood) and a short telomere IC (DNA extracted from K562 bone marrow lymphoblast cell cultures). For the mtDNA content assay, the positive ICs consisted of a high mtDNA IC (DNA extracted from SKBR3 mammary gland epithelial cell cultures) and a low mtDNA IC (DNA extracted from pooled whole blood). The inter-assay variability was 2.6% and 4.3% for the long and short telomere ICs, respectively, and 5.7% and 9.8% for the high and low mtDNA ICs, respectively. The rLTL was represented by the ratio of the telomere fluorescent signals normalized to the fluorescent signals of a single-copy nuclear gene (T/S ratio). The mtDNA content was defined as the copy number of mtDNA normalized to the copy number of a single-copy nuclear gene (mtDNA/nDNA ratio). Data for a specimen are accepted if the ratios of the duplicate measurement vary by <15%. If the duplicates fail this quality control criteria, they are run again. If the duplicates fail after being repeated, the mean and standard deviation of all four ratios are taken, and the standard deviation between ratios must be <10% for the mean of the four ratios to be accepted as the ratio for the specimen. The equations used to calculate the primary outcomes are presented in Appendix A (Table A1).

### 2.5. Statistical Analysis

A generalized linear model with adjustments for age and village cluster was also used to compare the GSH/GSSG ratio between women in the iron and placebo groups at 12 weeks. Wilcoxon rank-sum tests were used to compare rLTL and mtDNA values between groups, as well as to compare the unadjusted absolute and percent change in these biomarkers. Wilcoxon signed-rank tests were used to compare rLTL and mtDNA values within groups. Generalized linear mixed-effects models (intention-to-treat) were also used to predict the primary outcomes: the adjusted absolute and percent change in rLTL and mtDNA content among the iron and placebo groups after 12 weeks. We also conducted a per-protocol analysis that only included adherent women (those women who consumed ≥80% of the allocated capsules). The rLTL models were adjusted for age and baseline rLTL (fixed-effects) and village clusters (random-effects). Age was adjusted for as it is inversely associated with LTL [44]. Similarly, baseline rLTL was adjusted for as telomere length is reported to be positively associated with the rate of telomere attrition [44]. The mtDNA content model was adjusted for age (fixed-effect) and village clusters (random-effects). Age was adjusted for as it is reported to be inversely associated with mtDNA content [45]. We also tested for an interaction effect between baseline iron status (ferritin concentration) and iron supplementation on both rLTL and mtDNA content outcomes. Ferritin concentrations were adjusted for inflammation based on levels of C-reactive protein (CRP) and α-1-acid glycoprotein (AGP) [46]. We also tested for an interaction effect between the presence of a genetic hemoglobin disorder and iron supplementation on rLTL and mtDNA content. A *p*-value of α < 0.05 was considered statistically significant. All analyses were conducted using Stata IC v.15.1 (Stata Corp., College Station, TX, USA).

## 3. Results

### 3.1. Baseline Characteristics

Descriptive characteristics and baseline indicators for these women are presented in Table 1. Baseline anemia prevalence was high (52–60%), yet prevalence of iron deficiency based on a ferritin concentration of <15 µg/L was low (20–25%). Genetic hemoglobin disorders were common, affecting the majority of the women.

### 3.2. GSH/GSSG Ratio

In our pilot study of *n* = 69 randomly selected women, we observed a trend for higher oxidative stress in women in the iron group (indicated by a lower GSH/GSSG ratio), as compared with placebo (ß: −8.1 [95% CI: −17.5, 1.3]; *p =* 0.092). Based on these initial findings, we next measured two markers of cellular damage (rLTL and mtDNA content) among the full study sample (*n* = 376).

### 3.3. Baseline and 12-Week rLTL and mtDNA Content

Of the *n* = 377 women who completed the trial, baseline and 12-week samples from *n* = 376 women were analyzed for rLTL and mtDNA content. We determined rLTL for *n* = 376 women and mtDNA content for *n* = 370 women, as *n* = 6 specimens failed to meet the quality control criteria for the mtDNA content assay, as described in Section 2.3. The median (IQR) unadjusted baseline and 12-week values are presented in Table 2. No differences were found between the iron and placebo groups for baseline and 12-week rLTL and mtDNA content. Within group differences were observed in the iron group that suggested a significant decrease from baseline to 12-week rLTL (*p =* 0.003). An increase from baseline to 12-week mtDNA content was also observed in the placebo group (*p* < 0.001).

### 3.4. Unadjusted Change in rLTL and mtDNA Content after 12 Weeks

The unadjusted change in rLTL and mtDNA content after 12 weeks is shown in Table 3. The absolute and percent unadjusted change in rLTL over the 12-week supplementation period did not significantly differ between the iron and placebo groups. In contrast, a significant difference was observed for unadjusted absolute, but not percent, change in mtDNA content after 12 weeks, with a greater increase in the placebo group (*p =* 0.03).

### 3.5. Adjusted Change in rLTL and mtDNA Content after 12 Weeks

Table 4 shows the effect of iron supplementation on the change in rLTL and mtDNA content after 12 weeks. The stated findings refer to both absolute and percent change, unless specified. Iron supplementation was not significantly associated with the adjusted change in rLTL after 12 weeks, compared with placebo, among all women. This remained true in a sub-analysis of only adherent women. Age was significantly negatively associated with adjusted absolute and percent change in rLTL for the models that included all women (ß-coefficient: −0.01 [95% CI: −0.018, −0.002], *p =* 0.01; all ß-coefficient: −0.14 [95% CI: −0.256, −0.022], *p =* 0.02) and only adherent women (ß-coefficient: −0.01 [95% CI: −0.022, −0.004], *p =* 0.01; ß-coefficient: −0.17 [95% CI: −0.301, −0.0472], *p =* 0.01), such that older age was associated with a smaller increase or larger decrease in LTL change. Similarly, baseline rLTL was significantly associated with the adjusted change in rLTL for the models that included all women and only adherent women (*p* < 0.001). While mtDNA content increased in both groups after 12 weeks, the magnitude of the increase associated with iron supplementation was significantly smaller, compared with placebo, among all women and among only adherent women.

### 3.6. The Interaction Effect of Baseline Iron Status or Presence of a Genetic Hemoglobinopathy and Change in rLTL or mtDNA Content

Baseline serum ferritin concentration did not significantly modify the effect of iron supplementation on the adjusted change in rLTL (*p =* 1.00 and *p =* 0.99) or mtDNA content (*p =* 0.84 and *p =* 0.71) after 12 weeks. Similarly, the presence of any genetic hemoglobinopathy did not modify the effect of iron supplementation on the adjusted change in rLTL (*p =* 0.97 and *p =* 0.95) or mtDNA content (*p =* 0.09 and *p =* 0.19) after 12 weeks.

### 3.7. Prevalence of Anemia and Iron Deficiency at 12 Weeks

At 12 weeks, anemia prevalence was 42% (*n* = 81/191) in the iron group and 58% (*n* = 108/186) in the placebo group, and iron deficiency based on low ferritin concentrations was 1% (*n* = 2/190) in the iron group and 26% (*n* = 48/184) in the placebo group.

## 4. Discussion

This is the first study, to the best of our knowledge, to assess longitudinal changes in rLTL and mtDNA content in response to oral iron supplementation, based on data from a rigorous randomized controlled trial. In our sample, unadjusted rLTL was on the higher end of what has been previously reported [47,48,49]. It is difficult to compare our rLTL values to the current published literature owing to inter-laboratory variation in pre-analytical and analytical factors [50], as well as differences in the studied populations. Unadjusted mtDNA content was similar to another report [51], although there is still large variability in the data. While we found no association between iron supplementation and rLTL, we interestingly observed that iron supplementation was associated with a significantly smaller increase in mtDNA content after 12 weeks compared with placebo. This finding was unexpected and, unfortunately, we do not have the data needed to explore this further. However, we speculate that it is unlikely that iron was providing a protective effect against some external environmental factor, given its pro-oxidant nature [18]. Our pilot data support this assumption, given that the GSH/GSSG ratio was lower in women who received iron as opposed to placebo, suggesting elevated oxidative stress in this group.

Priliani et al. [39] recently reported that pregnant Indonesian women who consumed iron and folic acid (IFA) supplements for a median of 33 days had higher post-supplementation mtDNA content compared with women who consumed maternal multiple micronutrients over the same period. The increase in mtDNA content among women who consumed IFA was observed both during pregnancy and at three months postpartum. This effect during pregnancy may be the result of compensatory mechanisms for the putative mtDNA damage caused by iron exposure, as energy demand and oxidative stress increase with gestational age. Among both supplementation groups, the change in mtDNA content from baseline to post-supplementation was approximately 4 units. In comparison, the baseline to 12-week unadjusted change in mtDNA content for the iron and placebo groups in our study was 8 and 15 units, respectively. As our study only included non-pregnant women, we are limited in our ability to compare our results to the Priliani et al. trial. Furthermore, differences in the duration of supplementation and/or the time point of post-supplementation blood collection for mtDNA measurement may also be reasons for inconsistent findings across studies.

Similar to the findings described above, some cross-sectional studies have demonstrated a positive association between iron status or exposure and mtDNA content [28,52]. Data in these studies were collected at one time point, which does not capture changes in mtDNA content longitudinally [36]. Iron-induced mtDNA damage could at first result in mitophagy or halting of mitochondrial biogenesis, but over time could lead to mitochondrial biogenesis as the mitochondria try to compensate for the loss of mitochondria. Further, iron levels of the participants in these studies were much higher than those of the women in our study. In particular, Lal et al. [52] included subjects with transfusion-dependent thalassemia, a genetic disorder associated with altered iron metabolism and iron overload. More research is needed to determine how substantially higher baseline iron levels affect mitochondrial homeostasis. Differences in analysis could also explain the discrepancies in our results and the published literature. Lal et al. [52] did not have an age-matched control group, nor was age adjusted for when comparing the mean mtDNA content between the thalassemic subject group (4−53 years) and the control group (19–46 years). Given that age is associated with decreased mtDNA content [45], the age imbalance between the groups could affect the reported effect of iron exposure. In contrast, Kim et al. found no significant association between serum ferritin concentration and mtDNA content (ß-coefficient: −0.056, *p =* 0.06) in a model that was adjusted for multiple factors, including age [53]. In a recent cross-sectional study, Yu et al. [54] reported a negative association between iron intake and mtDNA content in men, but not women. This may have been owing to significantly higher reported iron intake and lower blood mtDNA content in men compared with women. Sex-specific differences may also be attributed to menstruation, which can lead to iron losses [55].

Despite previous cross-sectional studies demonstrating a negative association between iron status or iron supplementation and rLTL [56,57,58,59,60], we are the first to evaluate its effect longitudinally. Our findings show that iron supplementation with 60 mg of elemental iron for 12 weeks is insufficient to alter LTL. As with mtDNA content, discrepancies in our rLTL observations and the published observational literature may be related to differences in study design and population characteristics. Our studied population had a comparably lower iron status (e.g., ferritin concentration and/or transferrin saturation [TSAT]) compared with the populations of the aforementioned cross-sectional studies. However, past research has demonstrated that iron supplementation regimes similar in dose and duration have the potential to elicit non-transferrin bound iron (NTBI) production and biomarkers of oxidative stress in both iron-replete and iron-deplete women with unsaturated transferrin [61,62,63]. It is possible that NTBI and oxidative stress markers are sub-clinical indicators of iron-mediated damage that are insufficient to alter LTL. A sub-analysis among 100 randomly selected women in our study revealed that only *n* = 17 had an elevated serum NTBI (≥0.1 μmol/L) after 12 weeks of supplementation, but this did not appear to be associated with iron supplementation (*n* = 9 were in the Fe group and *n* = 8 were in the placebo group) [64]. However, this sub-analysis may have been underpowered owing to a small sample size and the late timing of blood collection may have hindered the ability to observe the peak of NTBI in serum. Additionally, our pilot data indicate this iron supplementation regime may be associated with increased oxidative stress. However, it is important to note that the use of the GSH/GSSG ratio to reflect redox is debated and any fluctuations may be reflective of other metabolic dysfunction [65]. We also note that our plasma specimens from the 2015 trial were not pre-treated prior to being frozen at −80 °C; specimens to be used for determining GSH/GSSG are often treated with different agents to prevent artifactual autoxidation of GSH, which may lead to the overestimation of oxidative stress [24]. However, we note that that Arbor Assay test kit did not specify pre-treatment with reducing agents was required.

Higher doses, durations, and adherence increase iron exposure and can cause more oxidative stress, which may explain why Xu et al. [60] observed that women who took iron supplements had significantly shorter LTL compared with those who did not. Further, different types of iron supplements vary in bioavailability and in the magnitude of oxidative stress produced [66,67,68]. Low mean daily iron intake, which would not be expected to cause oxidative stress in healthy individuals, may explain why Lee et al. [69] observed no association between iron intake and LTL. Similarly, low mean daily iron and high vitamin C intakes could be the reason Mazidi et al. [70] observed a positive association between iron intake and LTL. Vitamin C is a powerful antioxidant, which has the potential to mitigate oxidative stress that could be caused by excess iron intake [71,72].

As our study sample contained a mix of both iron-deplete and iron-replete women at baseline, we assessed the interaction effect between baseline serum ferritin concentration and iron supplementation on both rLTL and mtDNA content at 12 weeks. No interaction was observed, suggesting that the effect of iron supplementation on these outcomes was independent of the presence or absence of iron deficiency at baseline. We similarly observed no interaction effect between the presence of a genetic hemoglobin disorder and iron supplementation on rLTL and mtDNA content. Future studies should also consider these factors as they may impact policy decisions regarding untargeted supplementation regimes.

There are no known published studies that have investigated the effect of 60 mg of elemental iron for 12 weeks on the change in rLTL or mtDNA content. The dose and duration of exposure to iron supplements that may lead to the damage of telomeric DNA are not yet clear. The rigorous study design of our randomized controlled trial is better suited to measure changes in rLTL and mtDNA content that are a result of iron supplementation, as compared with cross-sectional studies that can only infer association. Further, the MMqPCR assay used in our study for the rLTL and mtDNA content analyses is a robust high-throughput method that has been validated against other methods of telomere length and mtDNA content measurement [43]. Some limitations of our study include a short duration of supplementation (perhaps not long enough to observe the effect of iron on rLTL) as well as a lack of other measured external factors, which could have contributed to a more comprehensive interpretation of our findings. Given the potential danger of excessive iron, and the current knowledge gap around the effect of untargeted supplementation, rigorous experimental studies and biomarkers are needed.

## 5. Conclusions

In conclusion, it is likely that 60 mg/d of elemental iron as ferrous sulfate for 12 weeks does not affect rLTL, but is associated with altered mitochondrial homeostasis. More research is needed to understand the effect of iron supplementation on mitochondrial homeostasis and potential associated health consequences.

## Figures and Tables

**Table 1:** Baseline indicators and prevalence rates for women by supplement group ^1^.

	Iron	Placebo
Women included in analysis	190 (51%)	186 (49%)
Age, years	31 ± 8	30 ± 8
Indicators		
Hb, g/L	116 ± 14	117 ± 13
Storage iron, ferritin ^2^, μg/L	39.1 (18.0, 81.4)	37.1 (15.1, 61.0)
Tissue iron, sTfR, mg/L	6.0 (4.8, 8.3)	5.9 (4.7, 7.7)
TSAT, %	22.5 (14.0, 29.3)	21.3 (12.3, 28.9)
CRP, mg/L	0.37 (0.18, 0.87)	0.43 (0.23, 1.03)
AGP, mg/L	0.55 (0.45, 0.72)	0.56 (0.46, 0.70)
Anemia prevalence		
Anemia, Hb < 120 g/L	114 (60%)	96 (52%)
IDA, ferritin ^2^ < 15 μg/L and Hb < 120 g/L	33/189 (17%)	38 (20%)
IDA, sTfR > 8.3 mg/L and Hb < 120 g/L	44/189 (23%)	36 (19%)
Iron deficiency prevalence		
Ferritin ^2^ < 15 μg/L	38/189 (20%)	46 (25%)
sTfR > 8.3 mg/L	48/189 (25%)	42 (23%)
Genetic hemoglobin disorder prevalence		
Any (Hb variant or α-thalassemia)	150 (79%)	130 (70%)
Hb variant (E, CS, H, Bart, or F)	115 (61%)	98 (53%)
α-thalassemia mutation	83/189 (44%)	76/185 (41%)
Inflammation prevalence		
Acute inflammation, CRP > 5 mg/L	9/189 (5%)	4 (2%)
Chronic inflammation, AGP > 1 g/L	15/189 (8%)	14 (8%)

^1^ Total *n* = 376. Values are mean ± SD, median (IQR), or *n* (%). Hb, hemoglobin; sTfR, soluble transferrin receptor; TSAT, transferrin saturation; CRP, C-reactive protein; AGP, α-1-acid glycoprotein; IDA, iron deficiency anemia. ^2^ Ferritin concentrations were adjusted for inflammation based on levels of CRP and AGP [46].

**Table 2:** Unadjusted baseline and 12-week rLTL and mtDNA content by supplement group ^1,2^.

	Iron	Placebo
Women included in rLTL analysis	190 (51%)	186 (49%)
Baseline rLTL	7.1 (6.5, 7.7)	7.1 (6.5, 7.8)
12-week rLTL	7.0 (6.4, 7.7)	7.1 (6.1, 7.8)
Women included in mtDNA analysis	186 (50%)	184 (50%)
Baseline mtDNA	95 (73, 120)	90 (65, 115)
12-week mtDNA	103 (83, 122)	105 (78, 135)

^1^ Values are median (IQR) or *n* (%). rLTL, relative leukocyte telomere length; mtDNA, mitochondrial DNA. ^2^ No significant differences were detected between groups at baseline or at 12 weeks. Significant within group differences from baseline to 12 weeks were found in the iron group for rLTL (*p =* 0.003) and in the placebo group for mtDNA content (*p* < 0.001) (Wilcoxon signed-rank tests).

**Table 3:** Unadjusted change in rLTL and mtDNA content after 12 weeks ^1,2^.

	Iron rLTL: *n* = 190 mtDNA: *n* = 186	Placebo rLTL: *n* = 186 mtDNA: *n* = 184	*p*
Change in rLTL after 12 weeks			
Absolute	−0.2 (−0.4, 0.2)	−0.1 (−0.5, 0.3)	0.34
Percent	−2.1% (−6.1%, 3.3%)	−1.4% (−5.9%, 4.8%)	0.29
Change in mtDNA after 12 weeks			
Absolute	3 (−18, 28)	11 (−7, 37)	0.03
Percent	6% (−18%, 35%)	12% (−11%, 47%)	0.07

^1^ Values are median (IQR). rLTL, relative leukocyte telomere length; mtDNA, mitochondrial DNA. ^2^ Differences between groups were assessed by Wilcoxon rank-sum test.

**Table 4:** Adjusted effect of iron supplementation on change in rLTL and mtDNA content after 12 weeks ^1^.

	All Women rLTL: *n* = 376 mtDNA: *n* = 370		Adherent Women rLTL: *n* = 325 mtDNA: *n* = 321	
	**ß (95% CI)**	***p***	**ß (95% CI)**	***p***
Change in rLTL after 12 weeks ^2^				
Absolute	−0.04 (−0.16, 0.08)	0.50	−0.01 (−0.14, 0.12)	0.86
Percent	−0.96 (−2.69, 0.77)	0.28	−0.53 (−2.41, 1.34)	0.58
Change in mtDNA after 12 weeks ^3^				
Absolute	−11 (−12, −2)	0.02	−12 (−22, −3)	0.01
Percent	−11 (−20, −1)	0.02	−12 (−22, −2)	0.02

^1^ CI, confidence interval; rLTL, relative leukocyte telomere length; mtDNA, mitochondrial DNA. ^2^ rLTL model adjusted for age and baseline rLTL (fixed effects) and village clusters (random effects). ^3^ mtDNA content model adjusted for age (fixed effects) and village clusters (random effects).

## Data Availability

Data are available upon request due to ethical and privacy restrictions.

## Appendix A

**Table A1:** Primary outcome equations ^1^.

Primary Outcome	Equation
Absolute change in rLTL	12-week rLTL− baseline rLTL
Percent change in rLTL	$\frac{12-\mathrm{week}\;\mathrm{rLTL}-\;\mathrm{baseline}\;\mathrm{rLTL}}{\mathrm{baseline}\;\mathrm{rLTL}}\times 100$
Absolute change in mtDNA Content	12-week mtDNA content− baseline mtDNA content
Percent change in mtDNA content	$\frac{12-\mathrm{week}\;\mathrm{mtDNA}\;\mathrm{content}-\;\mathrm{baseline}\;\mathrm{mtDNA}\;\mathrm{content}}{\mathrm{baseline}\;\mathrm{mtDNA}\;\mathrm{content}}\times 100$

^1^ rLTL, relative leukocyte telomere length; mtDNA, mitochondrial DNA.
